# Minimally Invasive Postmortem Intestinal Tissue Sampling in Malnourished and Acutely Ill Children Is Feasible and Informative

**DOI:** 10.1093/cid/ciab790

**Published:** 2021-12-15

**Authors:** Erika Feutz, Wieger Voskuijl, Peter J Finch, Ta-Chiang Liu, Robert H J Bandsma, Phillip I Tarr, Christopher Alan Moxon, Kelley VanBuskirk, Sarah Lawrence, Grace Umutesi, Kirkby D Tickell, James A Berkley, Judd L Walson, Steve Kamiza, Donna M Denno

**Affiliations:** 1 Department of Global Health, University of Washington, Seattle, Washington, USA; 2 Amsterdam Centre for Global Child Health, Emma Children’s Hospital, Amsterdam University Medical Centres, Amsterdam, The Netherlands; 3 Amsterdam Institute for Global Health and Development, Amsterdam University Medical Centres, Amsterdam, The Netherlands; 4 Department of Paediatrics and Child Health, Kamuzu University of Health Sciences, Blantyre, Malawi; 5 Department of Medicine, Kamuzu University of Health Sciences, Blantyre, Malawi; 6 Department of Pathology and Immunology, Washington University School of Medicine, St. Louis, Missouri, USA; 7 Hospital for Sick Children, Translational Medicine Program and Centre for Global Child Health, Toronto, Canada; 8 The Childhood Acute Illness & Nutrition (CHAIN) Network, Nairobi, Kenya; 9 Department of Pediatrics, Washington University School of Medicine, St. Louis, Missouri, USA; 10 Wellcome Centre for Integrative Parasitology, Institute of Infection, Immunity and Inflammation, College of Medical, Veterinary and Life Sciences, University of Glasgow, Glasgow, UK; 11 Malawi-Liverpool Wellcome Clinical Research Programme, College of Medicine, University of Malawi, Blantyre, Malawi; 12 Department of Pediatrics, University of Washington, Seattle, Washington, USA; 13 Department of Epidemiology, University of Washington, Seattle, Washington, USA; 14 KEMRI-Wellcome Trust Research Programme, Nairobi, Kenya; 15 Centre for Tropical Medicine & Global Health, University of Oxford, Oxford, UK; 16 Department of Medicine, University of Washington, Seattle, Washington, USA; 17 Department of Pathology, Kamuzu University of Health Sciences, Blantyre, Malawi

**Keywords:** Minimally invasive tissue sampling, environmental enteric dysfunction, histopathology, child mortality

## Abstract

**Background:**

Intestinal disorders such as environmental enteric dysfunction (EED) are prevalent in low- and middle-income countries (LMICs) and important contributors to childhood undernutrition and mortality. Autopsies are rarely performed in LMICs but minimally invasive tissue sampling is increasingly deployed as a more feasible and acceptable procedure, although protocols have been devoid of intestinal sampling to date. We sought to determine (1) the feasibility of postmortem intestinal sampling, (2) whether autolysis precludes enteric biopsies’ utility, and (3) histopathologic features among children who died during hospitalization with acute illness or undernutrition.

**Methods:**

Transabdominal needle and endoscopic forceps upper and lower intestinal sampling were conducted among children aged 1 week to 59 months who died while hospitalized in Blantyre, Malawi. Autolysis ratings were determined for each hematoxylin and eosin slide, and upper and lower intestinal scoring systems were adapted to assess histopathologic features and their severity.

**Results:**

Endoscopic and transabdominal sampling procedures were attempted in 28 and 14 cases, respectively, with >90% success obtaining targeted tissue. Varying degrees of autolysis were present in all samples and precluded histopathologic scoring of 6% of 122 biopsies. Greater autolysis in duodenal samples was seen with longer postmortem interval (Beta = 0.06, 95% confidence interval, 0.02–0.11). Histopathologic features identified included duodenal Paneth and goblet cell depletion. Acute inflammation was absent but chronic inflammation was prevalent in both upper and lower enteric samples. Severe chronic rectal inflammation was identified in children as young as 5.5 weeks.

**Conclusions:**

Minimally invasive postmortem intestinal sampling is feasible and identifies histopathology that can inform mortality contributors.

Undernutrition underlies 45% of child deaths and remains prevalent in low- and middle-income countries, including Malawi, where the under-5 year mortality rate is 42 per 1000 live births: 11% above the global average [1–3]. Causes of undernutrition include food insecurity, recurrent infections, and environmental enteric dysfunction (EED). A largely asymptomatic condition, EED is prevalent in settings with inadequate sanitation and hygiene facilities. It is characterized by intestinal inflammation and villus blunting, malabsorption, permeability facilitating microbe translocation, and systemic inflammation [4, 5].

Accelerating mortality reductions requires improved cause of death (COD) understanding, particularly within populations in which undernutrition and infections are the leading mortality risk factors [3, 6]. Full autopsy is the most comprehensive and accurate method for COD assignment, but is often infeasible because of resource constraints or societal unacceptance [7, 8]. Minimally invasive tissue sampling (MITS) uses transcutaneous needle organ and fluid sampling and is increasingly used as a validated, nondisfiguring, and more acceptable alternative to full autopsy [9, 10]. However, MITS lacks enteric sampling because intestines, largely untethered within the abdominal cavity, likely enable needles to push away, potentially hindering sampling of this organ. Endoscopy may afford visualization and sampling but has rarely been used, and to our knowledge, never attempted in pediatric postmortem studies [11]. Several potential barriers could preclude this approach, including jaw, pyloric sphincter or anal rigor mortis restricting intubation, and stomach contents or stool interfering with visualization and sampling. Tissue autolysis, driven by microbes and cellular enzymes, may render biopsies uninformative because intestine is the first organ to degrade postmortem [12].

Lack of gastrointestinal interrogation impedes understanding of intestinal infections, EED, and other enteric contributions to childhood deaths. The MITS in Malawi (MiM) study sought to assess postmortem endoscopic intestinal sampling feasibility, determine whether autolysis precludes enteric biopsy utility, and examine enteric histopathologic features among children who died during hospitalization with acute illness or undernutrition.

## METHODS

### Study Design, Setting, Participant Selection

MiM was conducted at Queen Elizabeth Central Hospital (QECH) in Blantyre, Malawi, a national referral and teaching hospital. MiM started as a substudy at the QECH site of the Childhood Acute Illness & Nutrition (CHAIN) study, which assessed risk factors for mortality among children hospitalized with acute illness or undernutrition in low- and middle-income countries [13]. Because of low enrollment and case fatality among CHAIN-enrolled patients, MiM recruitment expanded to 2 other QECH-based studies and the general pediatric wards. Exclusion criteria were: known terminal illnesses, congenital syndromes, injuries, surgical conditions, and age <1 week or >59 months. Recruitment was August 20, 2018–April 9, 2020.

Study staff approached parents/guardians of eligible children after a respectful period following death. Written informed consent was obtained from parents/guardians. Assistance with coffin purchase, transportation, and grief support was offered to all approached parents/guardians, regardless of consent to participation.

### Sampling Procedures

MITS procedures commenced as soon as feasible after obtaining consent. A study gastroenterologist or endoscopist used Olympus GIF-P140, GIF XP-160, or GIF-160 gastroscopes and Olympus FB-231K.A or FB-230.K.A forceps. Gastric and duodenal fluid were aspirated, either prebiopsy (preferred) or postbiopsy after flushing with buffered saline. Biopsies were obtained from stomach, the first (D1), second (D2), and most distal accessible duodenal segments (D3/D4), rectum; and colon last as proximally as possible. Postendoscopy, standard MITS procedures were performed [14].

Transabdominal intestinal needle sampling was initially considered impractical, but was adopted midstudy to assess feasibility. Bard Monopty 16G 100-mm needles were introduced just inferior to the umbilicus and right-side posterolateral for small bowel and ascending colon samples, respectively. All tissues were paraffin embedded, sectioned, and stained with hematoxylin and eosin.

### Data Collection

Antemortem clinical data were collected during admission for coenrolled patients and otherwise extracted from medical records. Antemortem clinical test results were abstracted from laboratory reports. Because of incomplete antemortem anthropometric data, postmortem anthropometry was used, as is standard in MITS studies [15, 16]. Two study personnel measured mid-upper arm circumference, weight, and length and where discrepant, measured a third time. The average of 2 closest measurements was used. *Z* scores were calculated using World Health Organization Anthro software [17]. Severe acute malnutrition (SAM) was defined by mid-upper arm circumference <11.5 cm (among ≥6-month-old children), weight-for-length *z* score <-3, or nutritional edema [18, 19].

A gastrointestinal pathologist (T-C.L.) rated each intestinal hematoxylin and eosin slide for autolysis, categorized as: (0) no autolysis; (1) <50% of tissue autolyzed; (2) 50%–75%; (3) >75% but <100%; and (4) complete autolysis. All sufficiently intact duodenal slides were scored using the EED Biopsy Consortium histology index, whereby 10 histologic parameters are assigned semiquantitative scores from 0 (normal) to 3 or 4 (severe pathology) [20]. Lower intestinal histology was similarly scored based on 4 ulcerative colitis histological parameters [21]. Because slide preparation quality or autolysis may render certain features unscorable, we calculated total score percent (total score divided by maximum possible among scored parameters) to describe overall histopathologic severity [20].

### Data Analysis

Analysis was primarily descriptive because this study was designed to examine the feasibility of adding intestinal sampling to standard MITS procedures and not powered to comprehensively test associations with intestinal autolysis or histopathology. However, once feasibility was determined, we leveraged obtained tissues to explore such associations.

We examined autolysis variability by anatomic location and sampling method (endoscopic vs transabdominal) using paired *t* tests. Autolysis and histologic score intraindividual variability was determined with intraclass correlation coefficients (ICC) using a 2-way mixed effects model. We used univariate linear regression to explore the effect on autolysis by age, sex, admission diagnosis (sepsis, gastroenteritis), nutritional status (per postmortem anthropometry), human immunodeficiency virus (HIV) status, exposure to antibiotics that are potentially more gut microbiome disruptive (ceftriaxone, ciprofloxacin) versus less so (amikacin, amoxicillin, flucloxacillin, fluconazole, gentamicin, metronidazole, penicillin, trimethoprim-sulfamethoxazole) [22–24], refrigeration duration, postmortem interval (PMI; time to intestinal sampling), and endoscopically visualized stomach contents (restricted to autolysis of D1 because of proximity). All bodies were refrigerated. Refrigeration time highly correlates with PMI, so refrigeration time was calculated as percentage of PMI. Because the PMI/refrigeration duration and autolysis relationship was of primary a priori interest, they were assessed together in a multivariate model.

We used univariate linear regression to explore influences on histologic severity by age, sex, admission diagnosis (sepsis, gastroenteritis, anemia [can be caused by malabsorption]), HIV status, nutritional status, length of hospital stay, higher dysbiotic potential antibiotics (described previously), and hepatic steatosis (indicator of severe malnutrition).

Data were insufficient to relate blood culture results, blood gas acidosis, antibiotics (any use), and stomach pH to autolysis, and malaria diagnosis and acidosis to histologic severity. Analyses were conducted using Stata/SE 16.1. Significance was defined by 2-tailed alpha = .05. Graphics were generated using RStudio version 1.2.5019.

### Ethical Approval

The Malawi National Health Sciences Research Committee (NHSRC 1913) and Oxford University Ethics Committee (OxTREC 34-16) provided ethical approval. The University of Washington Institutional Review Board exempted the study from review (STUDY00003689).

## RESULTS

Seventy-five children eligible for this study died on enrollment days. Reasons for not seeking consent included: inability to mobilize a key study member within an appropriate time (n = 8), lack of notification by ward staff (n = 5), or unknown reasons (n = 4). Of 58 caregivers approached, 29 (50%) consented. Reasons for refusal were: lack of perceived benefit (n = 9), preference to immediately take body home (n = 7), cultural/religious concerns (n = 5), poor relationship with healthcare staff (n = 2), consenters unavailable (n = 2), child too young (n = 1), or unknown reasons (n = 3). Table 1 summarizes participant characteristics.

**Table 1.** Characteristics of Study Participants (n = 29)

	Median (IQR) or N (%)
Age at death, wk	24 (12–55)
Female	15 (51.7%)
Admission diagnoses (based on clinician diagnosis)	
Sepsis	9 (31.0%)
Acute respiratory infection^a^	7 (24.1%)
Gastroenteritis	10 (34.5%)
Malaria (based on testing, n = 1 missing)	2 (7.1%)
HIV infection (based on testing, n = 1 missing)	7 (24.1%)
Severe acute malnutrition^b^	18 (62.0%) including 5 (17.2%) with edematous SAM
Antibiotic administration during hospitalization^c^	29 (100%)
Administration of higher dysbiotic potential antibiotics during hospitalization^d^	15 (51.7%)
Length of hospital stay, d	3 (1–6)
Postmortem interval, h^e^	7 (6–11)
Body refrigeration time as a percentage of postmortem interval	88 (71–93)
Milk or food contents in stomach (Among those who underwent endoscopy, n = 28)	18 (64.3%)

Abbreviations: HIV, human immunodeficiency virus; IQR, interquartile range; SAM, severe acute malnutrition.

^a^Pneumonia (n = 4), bronchiolitis (n = 1), both (n = 2).

^b^Based on postmortem anthropometric measurements and antemortem assessment of edema.

^c^Ciprofloxacin, ceftriaxone, penicillin, gentamicin, metronidazole, cotrimoxazole, flucloxacillin, amikacin, amoxicillin, fluconazole.

^d^Ciprofloxacin and ceftriaxone.

^e^Time from death to initiation of minimally invasive tissue sampling procedure.

### Procedure Feasibility

Upper and lower endoscopic sampling was attempted for all but the final case because of corona virus disease 2019-related circumstances. Upper and lower endoscopy mean (range) duration were 34.4 (20-57) and 10.5 (6-20) minutes, respectively.

**Figure 1.**
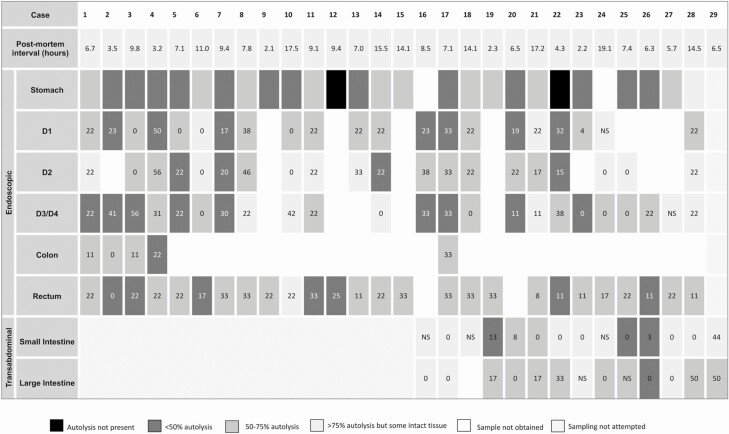
Samples of target tissue successfully obtained endoscopically and transabdominally by case. Transabdominal samples were attempted for cases 16–29. Endoscopic sampling was not attempted for case 29. Numbers in each cell represent intestinal histology disease severity as total score percent. Box color represents level of autolysis, with darker colors indicating less autolysis and better preserved tissue. Abbreviations: D1, D2, D3/D4: 1st, 2nd, 3rd/4th portions of the duodenum, respectively; NS, not scorable.

At least 1 intestinal biopsy was endoscopically obtained from each of 28 cases, including: 21 D1 (75%), 20 D2 (71%), 23 D3/D4 (82%), 5 colon (18%), and 26 rectum (93%) (Figure 1). Presence of stomach contents (eg, milk) and stool often caused insufficient equipment insufflation by procedure end. This explains colon sampling paucity because colon was the final sampling location, and twice interfered with rectal sampling. One case lacked duodenal sampling because of difficulty negotiating the C-loop. Pyloric sphincter constriction precluded duodenal intubation in 3 cases (PMIs between 2 and 14 hours). Transabdominal needle sampling of small and large intestine was successful in 14 (100%) and 13 (93%) of the last 14 cases, respectively. Across all participants, 26 (90%) and 29 (100%) had at least 1 successful small and large bowel biopsy, respectively, regardless of method.

### Tissue Autolysis

All intestinal samples displayed some autolysis (Figure 1, Supplementary Figure 1). Thirty-two biopsies (26%) had <50% autolysis, 59 (49%) had 50%–75%, and 31 (25%) had >75% autolysis. None were completely autolyzed. The mean autolysis rating was 1.9, corresponding to slightly <50%–75% of tissue autolyzed. In contrast, only 1 case demonstrated extraintestinal tissue autolysis, in the brain. Autolysis grading was inconsistent across biopsies within the same individual (ICC = 0.16) (Figure 1), but did not differ by gut location (Table 2). Among cases with both sampling methods, mean autolysis rating was higher in transcutaneous (2.3, standard deviation [SD] = 0.8) than endoscopic biopsies (1.9, SD = 0.7) (*P* = .040).

**Table 2.** Mean Autolysis Rating and Mean Intestinal Disease Severity Score Percent by Tissue and Sampling Type

Sampling Method	Sample Location (Cases 1–28 N/ Cases 16–29 N^a^)	Mean Autolysis Rating^b^, Cases 1–28, (SD, Range)	Mean Autolysis Rating^b^, Cases 16–29, (SD, Range)	Sample Location With Scorable Tissue (Cases 1–28 N/ Cases 16–29 N^c^) (Cases 1–28% Scorable/Cases 16–29% Scorable)^d^	Mean Histopathology Score Percent^e^, Cases 1–28, (SD, Range)	Mean Histopathology Score Percent^e^, Cases 16–29, (SD, Range)
Endoscopic	D1 (21/9)	1.8 (0.7, 1–3)	1.8 (0.8, 1–3)	D1 (20/9) (95/100)	19 (14, 0–50)	20 (11, 0–33)
	D2 (20/9)	2.2 (0.8, 1–3)	2.2 (0.7, 1–3)	D2 (20/9) (100/100)	21 (16, 0–56)	19 (13, 0–38)
	D3/D4 (23/12)	1.9 (0.8, 1–3)	1.9 (0.8, 1–3)	D3/D4 (22/11) (96/92)	21 (16, 0–56)	16 (15, 0–38)
	Colon (5/1)	1.8 (0.4, 1–2)	2 (…)	Colon (5/1) (100/100)	16 (13, 0–33)	33 (…)
	Rectum (26/11)	1.8 (0.5, 1–3)	1.8 (0.4, 1–2)	Rectum (26/11) (100/100)	21 (9, 0–33)	19 (10, 8–33)
Transabdominal	Small intestine (…/14)	…	2.4 (0.9, 1–3)	Small intestine (…/11) (.../79)	…	6 (13, 0–44)
	Large intestine (…/13)	…	2.2 (0.6, 1–3)	Large intestine (…/11) (.../85)	…	15 (20, 0–50)

^a^Endoscopic sampling was attempted for cases 1–28. Transabdominal sampling was attempted for cases 16–29. N represents the number of obtained biopsies successfully sampled and examined for autolysis.

^b^Autolysis ratings were coded as following: (0) no autolysis, (1) <50% of tissue autolyzed, (2) 50%–75%, (3) >75% but some intact tissue, and (4) complete autolysis.

^c^N represents the number of biopsies that could be examined for histopathologic features (ie, autolysis did not preclude scoring of at least 1 histologic feature).

^d^Some tissues were unable to be scored because extensive autolysis impeding the identification of histologic features.

^e^Autolysis rendered some histologic features nonscorable; therefore, total score percent (total score divided by maximum possible score of available criteria) is used to summarize histopathologic findings.

Upper intestinal autolysis was 0.4 points (out of 4) higher with a 6-hour increased PMI (95% confidence interval [CI]. 0.1–0.7, *P* = .01), without meaningful change after adjusting for refrigeration (as percent of PMI) (Table 3, Supplementary Figure 2). No clear relationship was appreciated between PMI and lower intestinal autolysis.

**Table 3.** Variables Univariately Tested for Association With Intestinal Tissue Autolysis Rating

	Beta Coefficients (95% CI) for Association With Autolysis in:					
	Endoscopically Sampled		Transabdominally Sampled		Combined	
	Upper Intestine^a^	Rectum	Small Intestine	Large Intestine	Upper Intestine^b^	Lower Intestine^c^
Age at death, wk	0.00 (-0.01 to 0.00)	0.00 (-0.01 to 0.00)	0.00 (0.00–0.01)	0.00 (0.00–0.00)	0.00 (0.00–0.01)	0.00 (0.00–0.00)
Female	0.45 (-0.05 to 0.95)	0.07 (-0.36 to 0.50)	0.46 (-0.54 to 1.46)	-0.19 (-0.95 to 0.57)	0.41 (-0.06 to 0.88)	0.01 (-0.43 to 0.44)
Sepsis admission diagnosis	-0.45 (-1.02 to 0.12)	0.51** **(0.11–0.92)**	-0.25 (-1.40 to 0.88)	0.39 (-0.40 to 1.18)	-0.44 (-0.97 to 0.09)	0.47* (0.04–0.90)
Gastroenteritis admission diagnosis	-0.48 (-0.97 to 0.02)	-0.16 (-0.59 to 0.27)	0.73 (-0.44 to 1.90)	0.57 (-0.26 to 1.39)	-0.39 (-0.88 to 0.10)	0.01 (-0.45 to 0.46)
HIV infection	-0.37 (-0.93 to 0.19)	-0.26 (-0.74 to 0.22)	-0.50 (-1.94 to 0.94)	-0.86 (-1.75 to 0.03)	-0.51 (-1.02 to 0.01)	-0.43 (-0.92 to 0.06)
Severe acute malnutrition (SAM)	-0.57* (-1.05 to -0.08)	-0.18 (-0.62 to 0.26)	-0.13 (-1.15 to 0.92)	0.12 (-0.64 to 0.88)	-0.58* (-1.03 to -0.13)	-0.24 (-0.68 to 0.20)
Edematous SAM	-0.14 (-0.79 to 0.51)	0.04 (-0.50 to 0.58)	0.80 (-0.22 to 1.83)	0.39 (-0.39 to 1.18)	0.15 (-0.48 to 0.78)	0.24 (-0.32 to 0.81)
Administration of higher dysbiotic potential antibiotics^d^	-0.51* (-1.00 to -0.03)	-0.31 (-0.71 to 0.10)	0.57 (-0.40 to 1.54)	0.12 (-0.66 to 0.88)	-0.35 (-0.83 to 0.13)	-0.20 (-0.62 to 0.22)
Postmortem time interval, h	0.07** (0.02–0.12)	0.03 (-0.01 to 0.07)	0.04 (-0.06 to 0.13)	-0.02 (-0.09 to 0.05)	0.06** (0.02–0.11)	0.01 (-0.03 to 0.06)
Postmortem interval, h, adjusted for refrigeration percentage	0.07** (0.01–0.13)	0.06* (0.01–0.11)	0.04 (-0.09 to 0.16)	-0.03 (-0.13 to 0.07)	0.07** (0.01–0.13)	0.03 (-0.02 to 0.09)
Refrigeration time (as percentage of postmortem time interval)	0.01 (-0.01 to 0.02)	0.00 (-0.01 to 0.00)	0.01 (-0.02 to 0.04)	0.00 (-0.01 to 0.01)	0.01 (0.00–0.02)	0.00 (-0.01 to 0.00)
Refrigeration (time as percentage of postmortem time interval), adjusted for postmortem time interval	0.01 (-0.01 to 0.02)	0.00 (-0.01 to 0.01)	0.01 (-0.02 to 0.04)	0.00 (-0.02 to 0.02)	0.01 (-0.01 to 0.02)	0.00 (-0.01 to 0.01)
Milk or food contents in stomach^e^	-0.51* (-1.01 to -0.08)	…	…	…	…	…

Bold text indicates statistically significant results (P < .05).

*P < .05.

**P < .01.

^a^Includes D1, D2, D3/D4, averaged by participant.

^b^Includes endoscopically sampled duodenal tissues (D1, D2, D3/D4) and transabdominally sampled small intestine, averaged by participant.

^c^ Includes endoscopically sampled rectum and transabdominally sampled large intestine, averaged by participant.

^d^Includes ciprofloxacin and ceftriaxone.

^e^Only tested for association with D1 because of proximity to gastric contents.

Rectal autolysis was 0.5 points higher among children diagnosed with sepsis (95% CI, 0.1–0.9, *P* = .032). Endoscopically-obtained small intestine autolysis was 0.6 points lower in those with SAM (95% CI, -1.1 to -0.1, *P* = .014). Antibiotic administration during hospitalization was universal, but those who received higher dysbiotic potential antibiotics had 0.5-point lower autolysis than those receiving other antibiotics, although this finding was limited to endoscopically obtained duodenal samples (95% CI, -1.0 to -0.03, *P* = .037). Those with endoscopically visible gastric contents had 0.5-point lower autolysis (only assessed in D1) (95% CI, -1.0 to -0.1, *P* = .031).

### Intestinal Histopathologic Disease Severity

Autolysis precluded scoring of 7 (6%) slides. At least 1 slide per case, totaling 115 slides (94%), were scored: 20 D1 (95%), 20 D2 (100%), 22 D3/D4 (96%), 5 colon (100%), and 26 rectal (100%) endoscopic biopsies and 11 transabdominal small (79%) and 11 large intestine samples (85%) (Figure 1, Table 2). Scores were inconsistent across tissues within individuals (ICC = 0.22 for all biopsies, ICC = 0.39 restricted to upper intestine only). Mean scores did not differ by enteric location (Table 2, Supplementary Figure 3). Transabdominally sampled small bowel had lower mean scores than endoscopically obtained (mean total score percent = 6% and 20%, respectively, *P* = .006).

Of upper intestinal histology features, Paneth cell depletion was most severely abnormal (mean score = 2 of 3), followed by chronic inflammatory infiltration (mean score = 1.8 of 3) and goblet cell depletion (mean score = 2 of 4) (Table 4, Supplementary Figure 4). Autolysis frequently precluded assessment of foveolar metaplasia, villus architecture, intraepithelial lymphocytes, and epithelial detachment. When assessable, mean scores were low (0, 0.7, 1, 1.3, and 1.5, respectively). Eosinophilic and neutrophilic infiltration were largely assessable, but uncommon (average scores 0.2 and 0, respectively). Lower intestine lacked evidence of neutrophilic infiltration or ulcerations (although the latter was infrequently assessable). Chronic rectal inflammation was prevalent and often severe (mean score = 2 of 3), including among 29% (n = 4) of infants younger than 6 months (Supplementary Figures 5 and 6). We explored factors that might have influenced this trend, but no relationships were identified. We found no relationships between a priori defined characteristics and histology scores.

**Table 4.** Number Scorable, Mean Score, and Range in Scores Within Criteria Used to Rate Intestinal Disease Severity

	D1 (n = 20)^d^			D2 (n = 20)			D3/D4 (n = 22)^d^			Transabdominal Small Intestine (n = 11)^d^			Rectum (n = 26)			Transabdominal Large Intestine (n = 11)^d^		
Histologic Parameter^a^ (Scored From 0–3 Except Where Noted)^b^	n	Mean	Range	n	Mean	Range	n	Mean	Range	n	Mean	Range	n	Mean	Range	n	Mean	Range
Acute inflammation	20	0	0–0	20	0	0–0	22	0	0–0	11	0	0–0						
Eosinophil infiltration	20	0	0–0	20	0	0–0	22	0	0–0	11	0.1	0–1						
Intraepithelial lymphocytes^c^	5	0.2	0–1	1	1	1–1	4	0.8	0–2	1	0	0–0						
Villus architecture^c^	3	1.7	1–2	0	…	…	1	2	2–2	1	0	0–0						
Foveolar metaplasia	3	0	0–0	0	…	…	2	0	0–0	1	0	0–0						
Goblet cell depletion^c^	5	2.2	1–4	3	2.3	1–4	4	2.5	1–4	2	0.5	0–1						
Paneth cell depletion	3	2	1–3	3	2	1–3	7	2.3	1–3	4	1.5	0–3						
Enterocyte injury	4	0.5	0–1	1	1	1–1	2	2	2–2	1	0	0–0						
Epithelial detachment^c^	5	1.6	0–3	2	1.5	0–3	3	2	1–3	1	0	0–0						
Lamina propria chronic inflammation	16	1.6	0–3	14	2.1	1–3	14	1.9	1–3	3	0	0–0						
Lamina propria neutrophils													26	0	0–0	10	0	0–0
Epithelial neutrophils													25	0	0–0	0	…	…
Chronic inflammation													26	2.0	0–3	9	1.1	0–3
Ulceration													3	0	0–0	0	…	…

^a^Scoring uses 10 criteria in upper intestinal tissues and 4 criteria in lower intestinal tissues.

^b^The environmental enteric dysfunction (EED) Biopsy Consortium initially published the use of 11 upper intestinal criteria; however, has since identified that the Brunner glands parameter has a negative relationship with EED, hence we excluded this parameter, but retained the other 10 original parameters [20].

^c^Possible scores range from 0 to 4.

^d^n represents the number of biopsies with at least one scorable parameter.

## Discussion

To our knowledge, this is the first pediatric use of minimally invasive intestinal sampling. In 22 adults from Hong Kong, Fan et al assessed postmortem laparoscopic and thoracoscopic approaches to various organs and endoscopy of stomach and great vessels. They also visualized (without biopsying) the duodenum endoscopically in 1 patient [25]. Postmortem upper endoscopy was attempted by Denzer et al in 20 German adults [11]. They reached D2 in 17 cases (stomach contents, pyloric atony, and anatomic issues precluded intestinal intubation in the others). However, intestinal sampling was only attempted (successfully) in 1 case. We are unaware of postmortem lower bowel endoscopy reports.

In our study, jaw rigor mortis did not inhibit oral intubation, though occasionally instrumented jaw opening was required. Anal rigor mortis, when present, was easily overcome. Pyloric sphincter rigor mortis impeded duodenal intubation in 3 cases. This phenomenon arises 2 hours postmortem and resolves by 12–14 hours in uncooled bodies [26]. It is encouraging that this obstruction was not more prevalent in this study, where most procedures commenced within 14 hours and all bodies underwent refrigeration. Stomach contents did not preclude endoscopic duodenal sampling. However, food/stool contents frequently blocked insufflation of endoscopy equipment by colon sampling at procedure end. We conclude that if a single endoscope is used per both oral and anal routes, routine colonic sampling is not feasible. Duodenal and rectal sampling is feasible, although needed equipment and clinical expertise may limit implementation to centers with requisite capacity.

The standard MITS procedure uses transcutaneous needle biopsies, but to our knowledge, this method had not been applied intestinally. We included transabdominal small and large bowel sampling for the last 14 cases with near universal success. Rapid needle firing likely facilitated intestinal tissue acquisition. Although likely more scalable than endoscopy, location within the upper intestine cannot be determined by blind biopsy nor can mucosa be grossly visualized. Preliminary data from an ongoing study suggest this approach unreliably yields samples in adults (unpublished). Moreover, preservation tended to be better in endoscopically sampled tissues than transabdominally. Therefore, endoscopic approach may be preferred, except in settings where precluded by resource strain.

Sampling utility is an important consideration even if feasible and scalable. Intestine autolyzes early. Microbial colonization is less abundant in the duodenum but likely an important driver of large bowel autolysis [12]. Digestive enzymes may be more involved in the duodenum, particularly D2 where pancreatic secretions drain. Although upper and lower intestine autolysis did not differ in this study, we noted a tendency toward tissue preservation with exposure to higher dysbiotic potential antibiotics (limited to small bowel). Less autolysis in children with SAM might be attributed to microbiome alterations or reduced pancreatic enzymatic activity associated with this condition [27, 28]. Less duodenal autolysis was found in the presence of stomach contents, perhaps because of protection from or competition for enzymatic degradation. Because sepsis was diagnosed by admitting clinician, its relationship with autolysis likely represents a relationship between illness severity at admission and autolysis rather than sepsis specifically.

Greater small bowel autolysis was seen with longer PMI, corroborating prior studies suggesting tissues become histologically uninformative beyond 12–24 hours [29–32]. Our data suggest that sampling is best performed within 6 hours postmortem as no tissues had ≥50% autolysis before this interval (Supplementary Figure 2). However, even samples obtained between 6 and 12 hours often yielded informative histology. Post-12 hours, no tissues had <50% autolysis.

Despite autolysis prevalence, many histologic features were discernible. As expected, epithelial structure was generally undiscernible because luminal surfaces degrade first. However, inflammatory response was scorable in 94% of slides. Duodenal chronic inflammation was prevalent. All tissues were devoid of acute inflammation. This is consistent with findings from biopsies of Zambian and Pakistani children with EED, as was our finding of reduced Paneth and goblet cell density which are important in antimicrobial activity and mucin production, respectively [20]. Depletion may be from rapid cell turnover or impaired stem cell differentiation [33]. EED histopathology investigations have focused on small bowel. Lower intestinal scrutiny has been limited. Rectal histology from this study demonstrated an absence of acute inflammation; however, chronic inflammation was identified in 96% of samples and severe infiltration seen in children as young as 5.5 weeks old. Although Chacko et al’s seminal autopsy investigation lacked inflammation assessment, they demonstrated generally normal fetal and neonatal villus architecture, whereas blunting was noted as early as age 8 weeks and severity increased with age [34]. Although our data are insufficient to explore further, factors including exposure to breastmilk substitutes or contaminated fluids could explain these findings. We hope to explore potential pathogen explanations in future analyses.

Transabdominally obtained small intestine had fewer histologic abnormalities than endoscopically obtained. Histopathology is a presampling phenomenon, so sampling method should not affect this. EED is thought to be a patchy duodenal disorder, as supported by our findings of intraindividual variation, possibly explained by sampling method difference. Because transabdominal sampling is blind, we cannot determine upper intestinal anatomic location and we may be scoring regions less affected than duodenum.

We hoped to explore relationships with histopathology to further elucidate the role of EED and intestinal pathology in child mortality. Though EED is considered an underlying cause of undernutrition, our study was unable to detect such relationship, likely from lack of statistical power [4]. Further, we did not identify relationships between histopathology and demographics, clinical interventions, or admission diagnoses. Pending final COD determination based on all MITS samples could enhance this exploration.

We acknowledge study limitations. Sample size was constrained because MiM was designed to assess feasibility; therefore, our assessment of relationships with autolysis and intestinal histopathology are exploratory. Additionally, participants died while at a referral hospital, where the majority of patients are from lower socioeconomic backgrounds and reside near Blantyre. Results may not generalize to community-based deaths or other populations.

Despite these limitations, our study demonstrates that minimally invasive postmortem intestinal sampling is feasible and histologically informative. Biopsy ascertainment within 12 hours of death appears to improve tissue preservation. Preservation may also be affected by patient characteristics (eg, nutritional status, clinical interventions). Histologic interrogation successfully identified and quantified features of EED and rectal chronic inflammation. Future studies with larger sample sizes should elucidate factors affecting autolysis and enteric disease and its role in child mortality and undernutrition.

## Supplementary Data

Supplementary materials are available at *Clinical Infectious Diseases* online. Consisting of data provided by the authors to benefit the reader, the posted materials are not copyedited and are the sole responsibility of the authors, so questions or comments should be addressed to the corresponding author.

ciab790_suppl_Supplementary_Figure_1

ciab790_suppl_Supplementary_Figure_2

ciab790_suppl_Supplementary_Figure_3

ciab790_suppl_Supplementary_Figure_4

ciab790_suppl_Supplementary_Figure_5

ciab790_suppl_Supplementary_Figure_6

ciab790_suppl_Supplementary_Figure_Legends

## Notes

***Acknowledgments.*** The authors gratefully thank the Childhood Acute Illness & Nutrition (CHAIN) Network and the Malawi site CHAIN team. The authors also thank Anstead Kankwatira, Queen Elizabeth Central Hospital endoscopist and gratefully acknowledge and appreciate the families who participated in this study.

***Supplement sponsorship.*** This supplement is sponsored by MITS Surveillance Alliance Secretariat, led by RTI International, with funding from the Bill and Melinda Gates Foundation.

***Financial support.*** This work was supported, in whole or in part, by the Bill & Melinda Gates Foundation (OPP1131320). Under the grant conditions of the Foundation, a Creative Commons Attribution 4.0 Generic License has already been assigned to the Author Accepted Manuscript version that might arise from this submission. J.A.B. reports research grants from MRC (UK) and Wellcome Trust paid to their institution.

***Potential conflicts of interest.*** The authors declare no conflicts of interest. J.A.B. reports being Chair 1 DSMB and 1 Trial Steering Committee for clinical trials in severely malnourished children outside Malawi. J.L.W. reports research grants from the Bill & Melinda Gates Foundation and National Institutes of Health paid to the institution outside of the conduct of the study. All authors have submitted the ICMJE Form for Disclosure of Potential Conflicts of Interest. Conflicts that the editors consider relevant to the content of the manuscript have been disclosed.

***Data availability.*** Underlying data available on Figshare (https://doi.org/10.6084/m9.figshare.14658906.v1)

## References

[1] UNICEF. Under-five mortality: child mortality data. Available at: https://data.unicef.org/topic/child-survival/under-five-mortality/. Accessed 03 January 2021.

[2] UNICEF. Malnutrition. Available at: https://data.unicef.org/topic/nutrition/malnutrition. Accessed 03 January 2021.

[3] Black RE, Victora CG, Walker SP, et al; Maternal and Child Nutrition Study Group. Maternal and child undernutrition and overweight in low-income and middle-income countries. Lancet 2013; 382:427–51. 23746772 10.1016/S0140-6736(13)60937-X

[4] Keusch GT, Denno DM, Black RE, et al. Environmental enteric dysfunction: pathogenesis, diagnosis, and clinical consequences. Clin Infect Dis 2014; 59(Suppl 4):S207–12. 25305288 10.1093/cid/ciu485 PMC4481570

[5] Watanabe K, Petri WA Jr. Environmental enteropathy: elusive but significant subclinical abnormalities in developing countries. EBioMedicine 2016; 10:25–32. 27495791 10.1016/j.ebiom.2016.07.030 PMC5006727

[6] United Nations Inter-agency Group for Child Mortality Estimation (UN IGME). Levels & trends in child mortality: report 2020, estimates developed by the United Nations Inter-agency Group for Child Mortality Estimation. New York, NY: United Nations Children’s Fund, 2020.

[7] Lewis C, Hill M, Arthurs OJ, Hutchinson C, Chitty LS, Sebire NJ. Factors affecting uptake of postmortem examination in the prenatal, perinatal and paediatric setting. BJOG 2018; 125:172–81. 28190300 10.1111/1471-0528.14600 PMC5763339

[8] Oluwasola OA, Fawole OI, Otegbayo AJ, Ogun GO, Adebamowo CA, Bamigboye AE. The autopsy: knowledge, attitude, and perceptions of doctors and relatives of the deceased. Arch Pathol Lab Med 2009; 133:78–82. 19123741 10.5858/133.1.78

[9] Lawrence S, Namusanya D, Hamuza A, et al. Hypothetical acceptability of hospital-based post-mortem pediatric minimally invasive tissue sampling in Malawi: the role of complex social relationships. PLoS One 2021; 16:e0246369. 33539411 10.1371/journal.pone.0246369 PMC7861399

[10] Bassat Q, Castillo P, Martínez MJ, et al. Validity of a minimally invasive autopsy tool for cause of death determination in pediatric deaths in Mozambique: an observational study. PLoS Med 2017; 14:e1002317. 28632739 10.1371/journal.pmed.1002317 PMC5478091

[11] Denzer UW, von Renteln D, Lübke A, et al. Minimally invasive autopsy by using postmortem endoluminal and transluminal endoscopy and EUS. Gastrointest Endosc 2013; 78:774–80. 24021488 10.1016/j.gie.2013.07.036

[12] Almulhim A, Menezes R. Evaluation of postmortem changes. Treasure Island, FL: StatPearls Publishing, 2021. 32119351

[13] The CHAIN Network. Childhood Acute Illness and Nutrition (CHAIN) Network: a protocol for a multi-site prospective cohort study to identify modifiable risk factors for mortality among acutely ill children in Africa and Asia. BMJ Open 2019; 9:e028454. 10.1136/bmjopen-2018-028454 PMC6502050 31061058

[14] Castillo P, Ussene E, Ismail MR, et al. Pathological methods applied to the investigation of causes of death in developing countries: minimally invasive autopsy approach. PLoS One 2015; 10:e0132057. 26126191 10.1371/journal.pone.0132057 PMC4488344

[15] Blau DM, Caneer JP, Philipsborn RP, et al. Overview and development of the child health and mortality prevention surveillance determination of cause of Death (DeCoDe) process and DeCoDe diagnosis standards. Clin Infect Dis 2019; 69:333–41. 10.1093/cid/ciz572 PMC6785670 31598661

[16] Paganelli C, Kassebaum N, Strong K, et al Guidance for systematic integration of undernutrition in cause of child death attribution. Clin Infect Dis 2021; 73(S5):S374–81. 10.1093/cid/ciab851 PMC8672773 34910171

[17] World Health Organization. WHO AnthroPlus for personal computers manual: software for assessing growth of the world’s children and adolescents. Geneva, Switzerland: World Health Organization, 2009.

[18] World Health Organization. Guideline: updates on the management of severe acute malnutrition in infants and children. Geneva, Switzerland: World Health Organization, 2013. 24649519

[19] World Health Organization. WHO child growth standards and the identification of severe acute malnutrition in infants and children: A joint statement by the World Health Organization and the United Nations Children’s Fund. Geneva, Switzerland: World Health Organization and UNICEF, 2009. 24809116

[20] Liu TC, VanBuskirk K, Ali SA, et al. A novel histological index for evaluation of environmental enteric dysfunction identifies geographic-specific features of enteropathy among children with suboptimal growth. PLoS Negl Trop Dis 2020; 14:e0007975. 31929525 10.1371/journal.pntd.0007975 PMC6980693

[21] Mosli MH, Feagan BG, Zou G, et al. Development and validation of a histological index for UC. Gut 2017; 66:50–8. 26475633 10.1136/gutjnl-2015-310393

[22] Bhalodi AA, van Engelen TSR, Virk HS, Wiersinga WJ. Impact of antimicrobial therapy on the gut microbiome. J Antimicrob Chemother 2019; 74:i6–i15. 30690540 10.1093/jac/dky530 PMC6382031

[23] Khan TJ, Hasan MN, Azhar EI, Yasir M. Association of gut dysbiosis with intestinal metabolites in response to antibiotic treatment. Hum Microb J 2019; 11:100054.

[24] Oldenburg CE, Sie A, Boubacar C, et al Effect of commonly used pediatric antibiotics on gut microbial diversity in preschool children in Burkina Fase: a randomized clinical trial. Open Forum Infect Dis 2018; 5:ofy289. 30515431 10.1093/ofid/ofy289 PMC6262116

[25] Fan JK, Tong DK, Poon JT, et al. Multimodality minimally invasive autopsy–a feasible and accurate approach to post-mortem examination. Forensic Sci Int 2010; 195:93–8. 20036088 10.1016/j.forsciint.2009.11.019

[26] Sudalaimuthu R, Manoharan C, Biradar BG, Shankar MB, Edwin JA. Role of pyloric sphincter in estimating time since death and in toxicological analysis. Indian J Forensic Community Med 2016; 3:96–100.

[27] Bartels RH, van den Brink DA, Bandsma RH, Boele van Hensbroek M, Tabbers MM, Voskuijl WP. The relation between malnutrition and the exocrine pancreas: a systematic review. J Pediatr Gastroenterol Nutr 2018; 66:193–203. 28991838 10.1097/MPG.0000000000001769

[28] Subramanian S, Huq S, Yatsunenko T, et al. Persistent gut microbiota immaturity in malnourished Bangladeshi children. Nature 2014; 510:417–21. 24896187 10.1038/nature13421 PMC4189846

[29] Creamer B, Leppard P. Post-mortem examination of a small intestine in the coeliac syndrome. Gut 1965; 6:466–71. 5321463 10.1136/gut.6.5.466 PMC1552328

[30] Cocariu EA, Mageriu V, Stăniceanu F, Bastian A, Socoliuc C, Zurac S. Correlations between the autolytic changes and postmortem interval in refrigerated cadavers. Rom J Intern Med 2016; 54:105–12. 27352439 10.1515/rjim-2016-0012

[31] Heimesaat MM, Boelke S, Fischer A, et al. Comprehensive postmortem analyses of intestinal microbiota changes and bacterial translocation in human flora associated mice. PLoS One 2012; 7:e40758. 22808253 10.1371/journal.pone.0040758 PMC3395637

[32] Damore LJ 2nd, Barth RF, Morrison CD, Frankel WL, Melvin WS. Laparoscopic postmortem examination: a minimally invasive approach to the autopsy. Ann Diagn Pathol 2000; 4:95–8. 10760323 10.1016/s1092-9134(00)90018-2

[33] Lueschow SR, McElroy SJ. The Paneth cell: the curator and defender of the immature small intestine. Front Immunol 2020; 11:587. 32308658 10.3389/fimmu.2020.00587 PMC7145889

[34] Chacko CJ, Paulson KA, Mathan VI, Baker SJ. The villus architecture of the small intestine in the tropics: a necropsy study. J Pathol 1969; 98:146–51. 5352370 10.1002/path.1710980209

