# Supplementary figures and images for: Minimally Invasive Postmortem Intestinal Tissue Sampling in Malnourished and Acutely Ill Children Is Feasible and Informative
Source: Clin Infect Dis. 2021 Dec 15;73(Suppl 5):S382–9. doi: 10.1093/cid/ciab790 (PMC8672761; doi:10.1093/cid/ciab790)

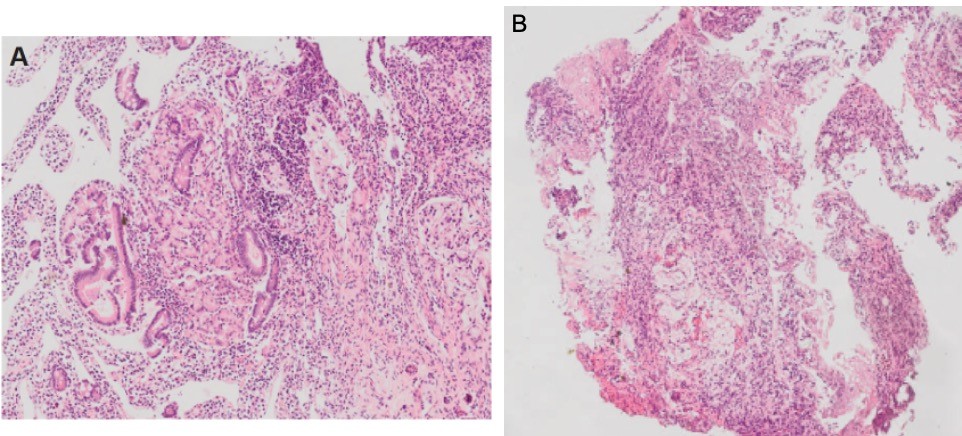

Supplement: ciab790_suppl_Supplementary_Figure_1 [file ciab790_suppl_supplementary_figure_1.jpeg]

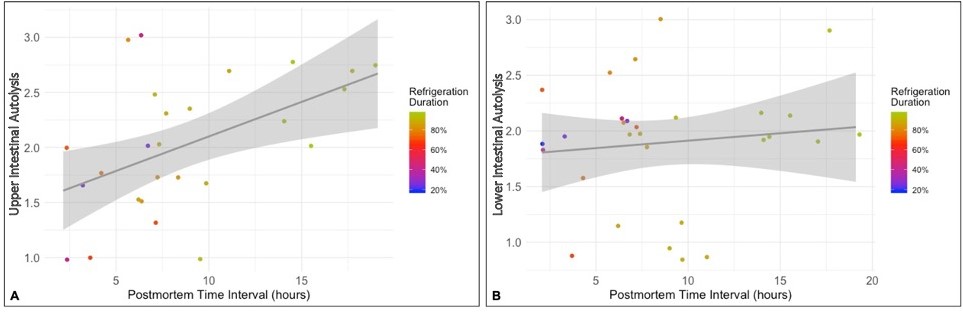

Supplement: ciab790_suppl_Supplementary_Figure_2 [file ciab790_suppl_supplementary_figure_2.jpeg]

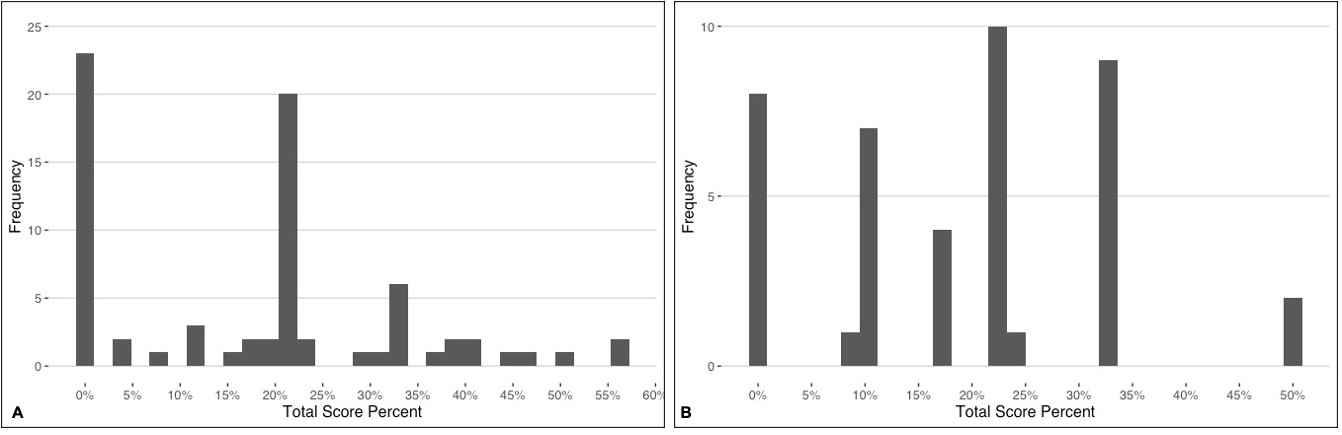

Supplement: ciab790_suppl_Supplementary_Figure_3 [file ciab790_suppl_supplementary_figure_3.jpeg]

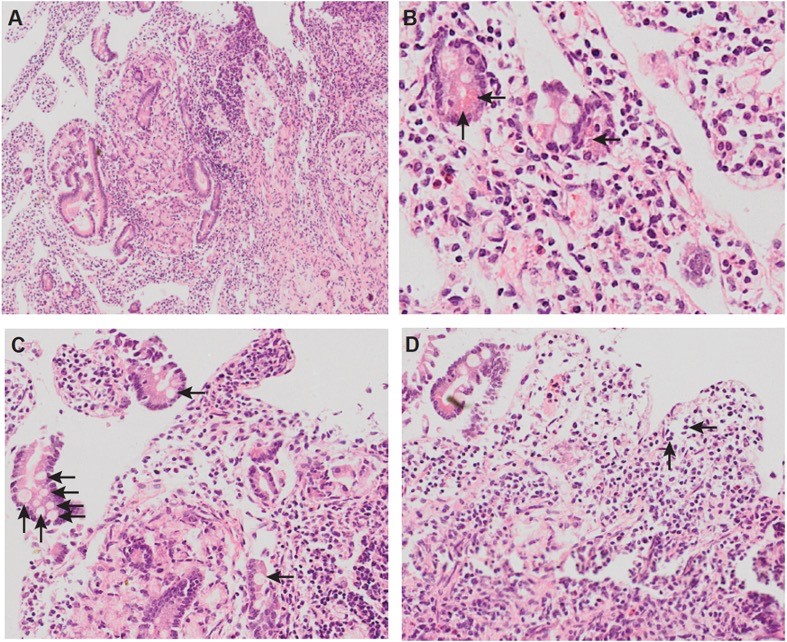

Supplement: ciab790_suppl_Supplementary_Figure_4 [file ciab790_suppl_supplementary_figure_4.jpeg]

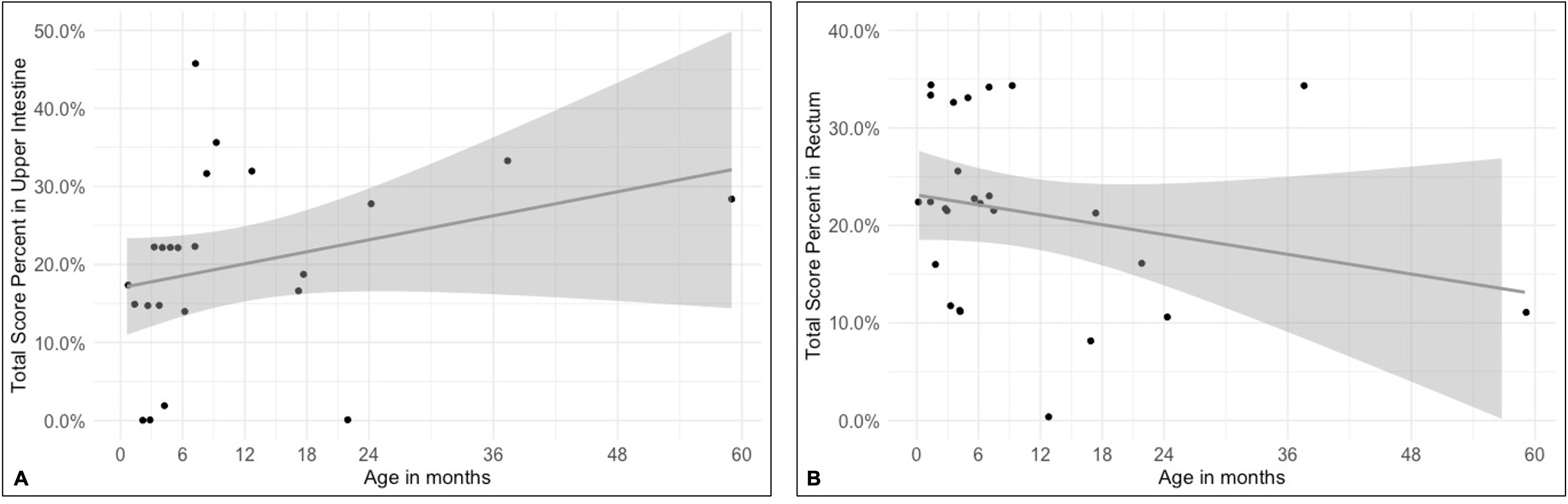

Supplement: ciab790_suppl_Supplementary_Figure_5 [file ciab790_suppl_supplementary_figure_5.jpeg]

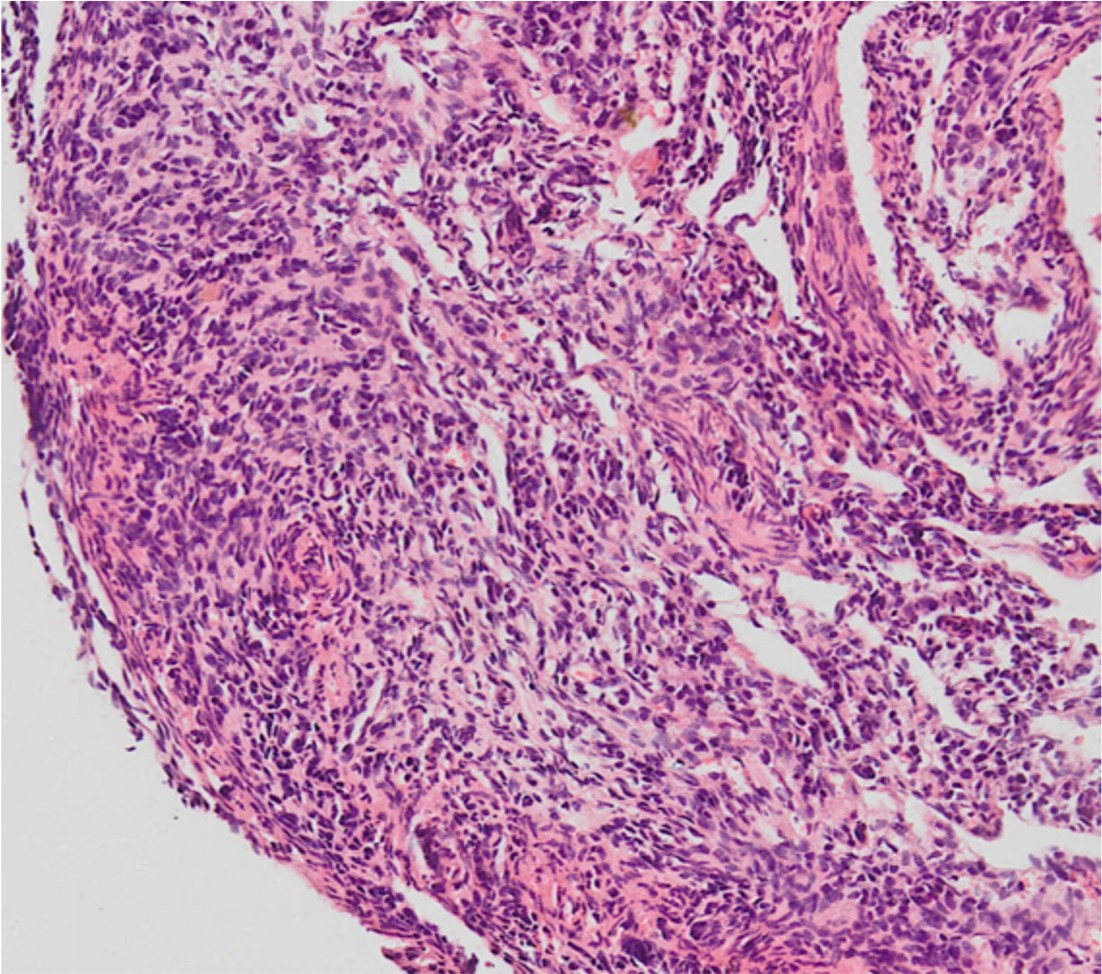

Supplement: ciab790_suppl_Supplementary_Figure_6 [file ciab790_suppl_supplementary_figure_6.jpeg]
