# Supplementary material for: Minimally Invasive Postmortem Intestinal Tissue Sampling in Malnourished and Acutely Ill Children Is Feasible and Informative
Source: Clin Infect Dis. 2021 Dec 15;73(Suppl 5):S382–9. doi: 10.1093/cid/ciab790 (PMC8672761; doi:10.1093/cid/ciab790)

**Supplementary Figure Legends:**

**Supplementary Figure 1:** **A** H&E-stained duodenal biopsy slide with minimal autolysis. **B** H&E-stained duodenal biopsy slide with extensive autolysis.

**Supplementary Figure 2:** Postmortem time interval and intestinal tissue autolysis. Colored points indicate refrigeration duration as a percentage of postmortem time interval. **A** Time from death to initiation of endoscopy, in hours, compared with average autolysis in upper intestinal biopsies (autolysis rating averaged across available intestinal locations). **B** Time from death to initiation of endoscopy, in hours, compared with autolysis rating in lower intestinal biopsies.

**Supplementary Figure 3:** Distribution of intestinal histopathology scores, as total score percent, in tissues obtained from the **A** upper intestine (endoscopically obtained D1, D2, and D3/D4 [1^st^, 2^nd^, and most distal accessible duodenal regions, respectively], and transabdominally obtained small intestine) and **B** lower intestine (endoscopically obtained colon and rectum, and transabdominally obtained large intestine).

**Supplementary Figure 4: A** A representative endoscopically obtained duodenal sample stained with H&E with low autolysis. **B** Paneth cells (arrows) are depleted in this sample. **C** Goblet cells (arrows) are not depleted in this sample. **D** Lamina propria lymphocytes (arrows) are abundant in this sample.

**Supplementary Figure 5:** Distribution of age and total intestinal disease severity total score percent for endoscopically obtained intestinal biopsies. **A** Age in months compared with total score percent in duodenal biopsies. **B** Age in months compared with total score percent in rectal tissue.

**Supplementary Figure 6:** H&E stained rectal biopsy slide from a 6-week old infant with extensive chronic inflammation (score=3).

**Supplementary Figures:**

**Supplementary Figure 1:**


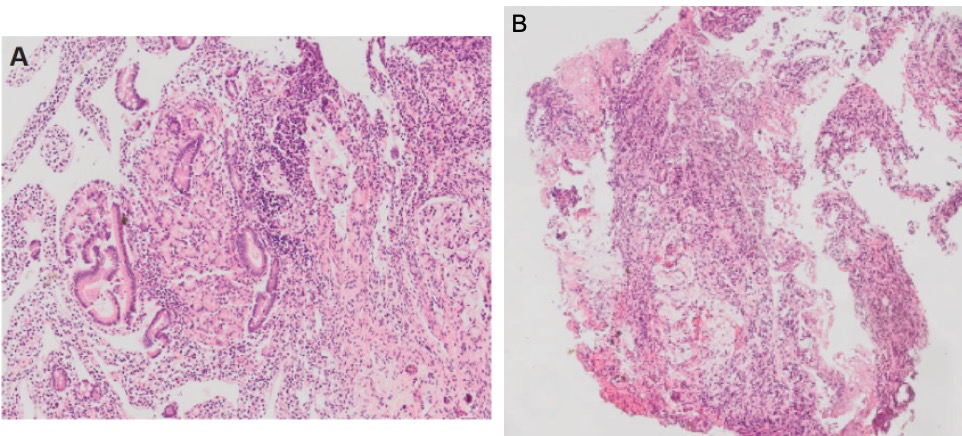


**Supplementary Figure 2:**


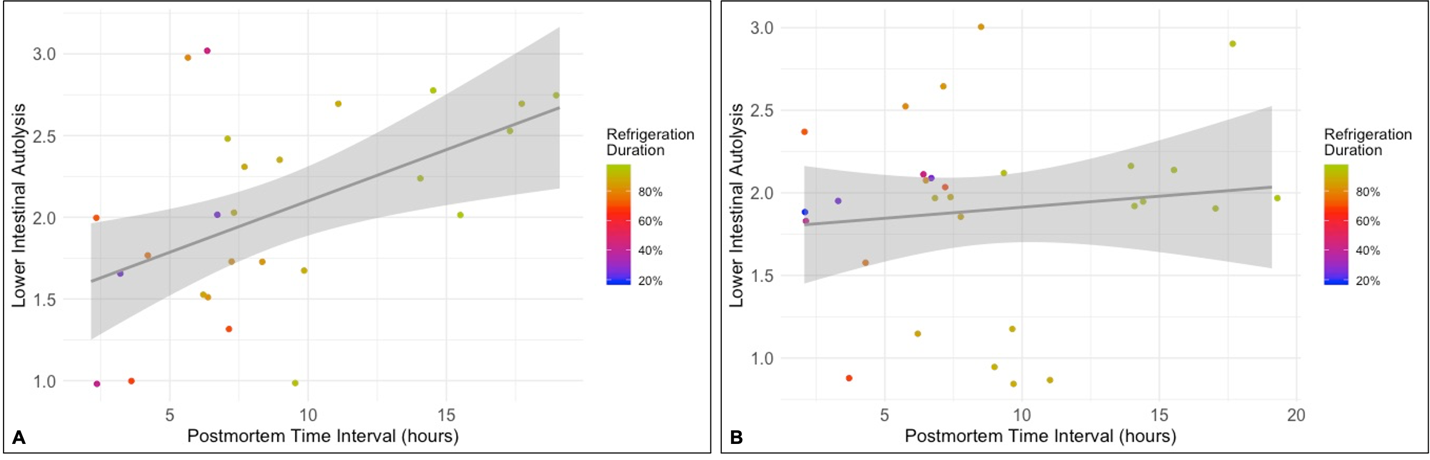


**Supplementary Figure 3:**


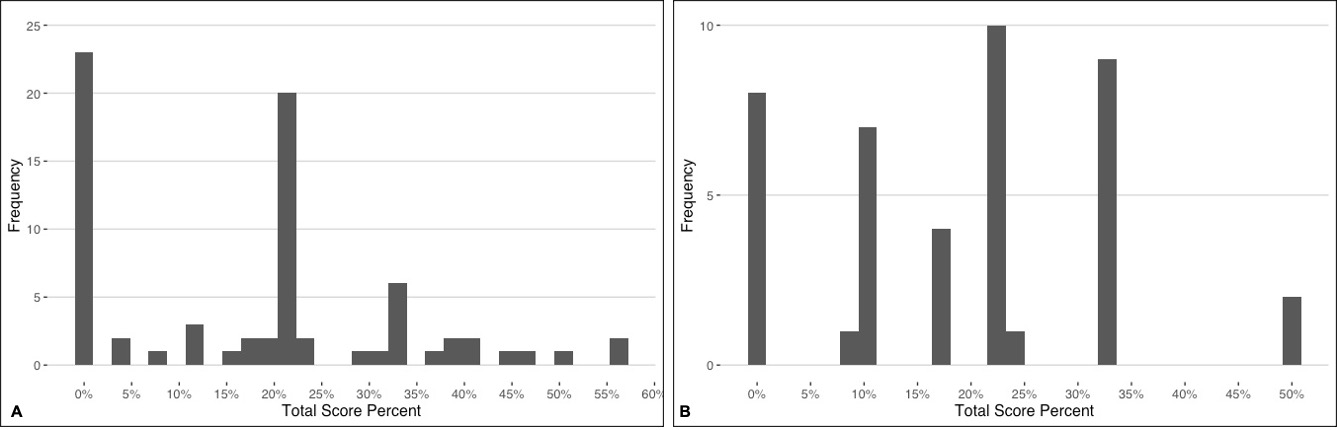


**Supplementary Figure 4:**


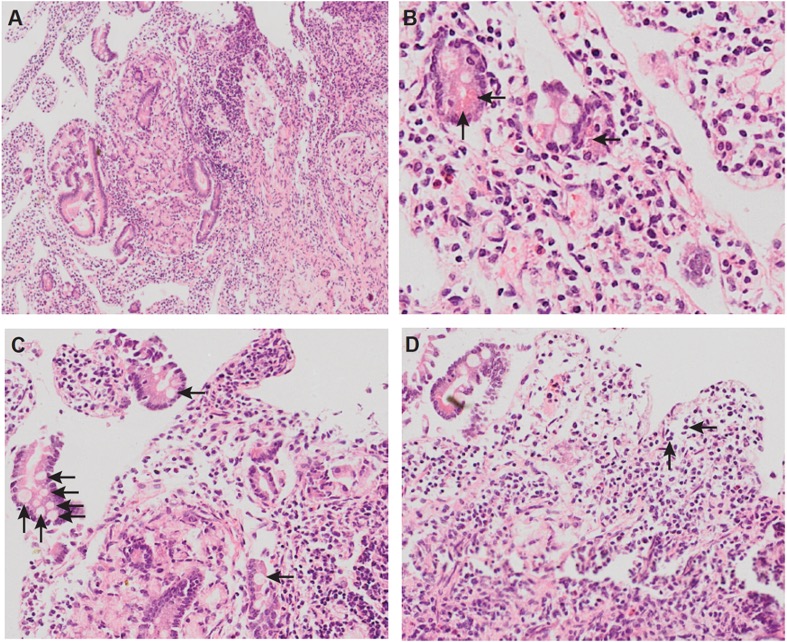


**Supplementary Figure 5:**


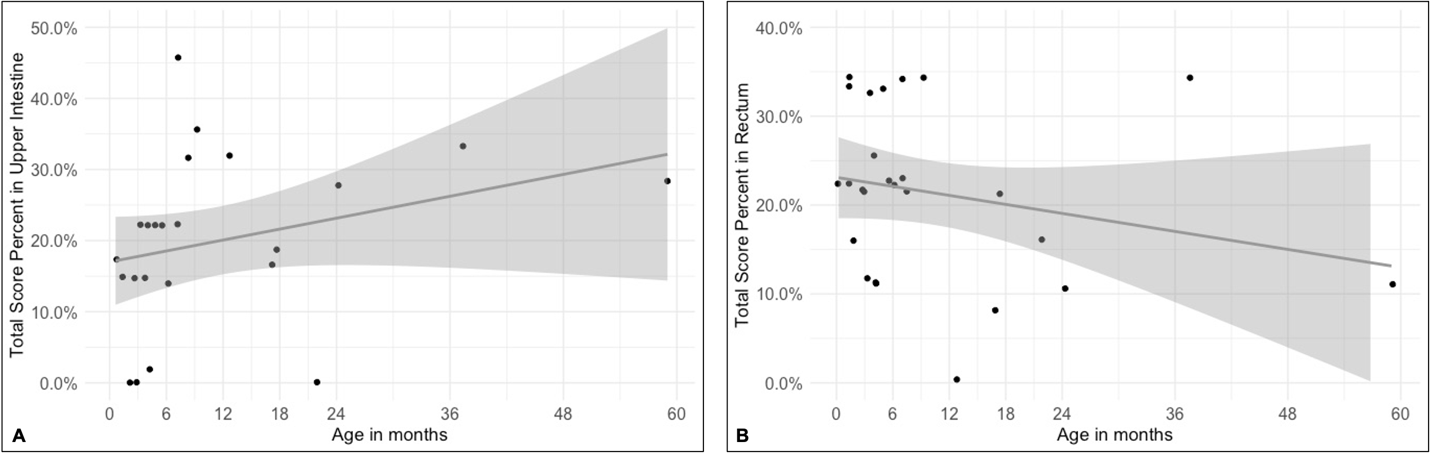


**Supplementary Figure 6:**


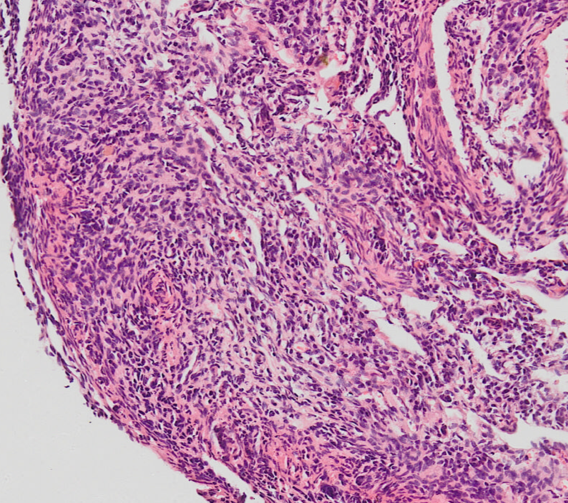

Supplement: ciab790_suppl_Supplementary_Figure_Legends [file ciab790_suppl_supplementary_figure_legends.docx]
